# Establishment and analysis of a novel mouse line carrying a conditional knockin allele of a cancer-specific *FBXW7* mutation

**DOI:** 10.1038/s41598-018-19769-1

**Published:** 2018-01-31

**Authors:** Tsuneo Ikenoue, Yumi Terakado, Chi Zhu, Xun Liu, Tomoyuki Ohsugi, Daisuke Matsubara, Tomoki Fujii, Shigeru Kakuta, Sachiko Kubo, Takuma Shibata, Kiyoshi Yamaguchi, Yoichiro Iwakura, Yoichi Furukawa

**Affiliations:** 1 Division of Clinical Genome Research, Advanced Clinical Research Center, The Institute of Medical Science, The University of Tokyo, Tokyo, Japan; 2 Department of Diagnostic Pathology, Jichi Medical University, Shimotsuke, Japan; 3 Department of Cancer Genome Research, Sasaki Institute, Sasaki Foundation, Tokyo, Japan; 4 Laboratory of Molecular Pathogenesis, Center for Experimental Medicine and Systems Biology, The Institute of Medical Science, The University of Tokyo, Tokyo, Japan; 5 Department of Biomedical Science, Graduate School of Agricultural and Life Sciences, The University of Tokyo, Tokyo, Japan; 6 Center for Animal Disease Models, Research Institute for Biomedical Sciences, Tokyo University of Science, Tokyo, Japan; 7 Division of Innate Immunity, Department of Microbiology and Immunology, The Institute of Medical Science, The University of Tokyo, Tokyo, Japan; 8 CREST, Japan Science and Technology agency, Kawaguchi, Japan

## Abstract

F-box and WD40 domain protein 7 (FBXW7) is a component of the SKP1-CUL1-F-box protein (SCF) complex that mediates the ubiquitination of diverse oncogenic target proteins. The exploration of FBXW7 mutations in human primary cancer has revealed three mutation hotspots at conserved arginine residues (Arg^465^, Arg^479^, and Arg^505^) in the WD40 domain, which are critical for substrate recognition. To study the function of human *FBXW7*^*R465C*^, the most frequent mutation in human malignancies, we generated a novel conditional knockin mouse line of murine *Fbxw7*^*R468C*^ corresponding to human *FBXW7*^*R465C*^. Systemic heterozygous knockin of the *Fbxw7*^*R468C*^ mutation resulted in perinatal lethality due to defects in lung development, and occasionally caused an eyes-open at birth phenotype and cleft palate. Furthermore, mice carrying liver-specific heterozygous and homozygous *Fbxw7*^*R468C*^ alleles cooperated with an oncogenic *Kras* mutation to exhibit bile duct hyperplasia within 8 months of birth and cholangiocarcinoma-like lesions within 8 weeks of birth, respectively. In addition, the substrates affected by the mutant Fbxw7 differed between the embryos, embryonic fibroblasts, and adult liver. This novel conditional knockin *Fbxw7*^*R468C*^ line should be useful to gain a more profound understanding of carcinogenesis associated with mutation of *FBXW7*.

## Introduction

FBXW7 (also known as FBW7, CDC4, SEL10, or AGO) is an F-box protein in the SKP1-CUL1-F-box protein (SCF) E3 ubiquitin ligase complex that controls the degradation of target substrates via the ubiquitin-proteasome pathway. Most FBXW7 substrates are positive regulators of cell growth, including c-MYC^1,2^, Cyclin E^3–5^, c-JUN^6^, NOTCH^7–9^, KLF5^10,11^, mTOR^12^, and MCL1^13,14^. FBXW7 has therefore been characterised as a tumour suppressor in human cancer.

**Figure 1:**
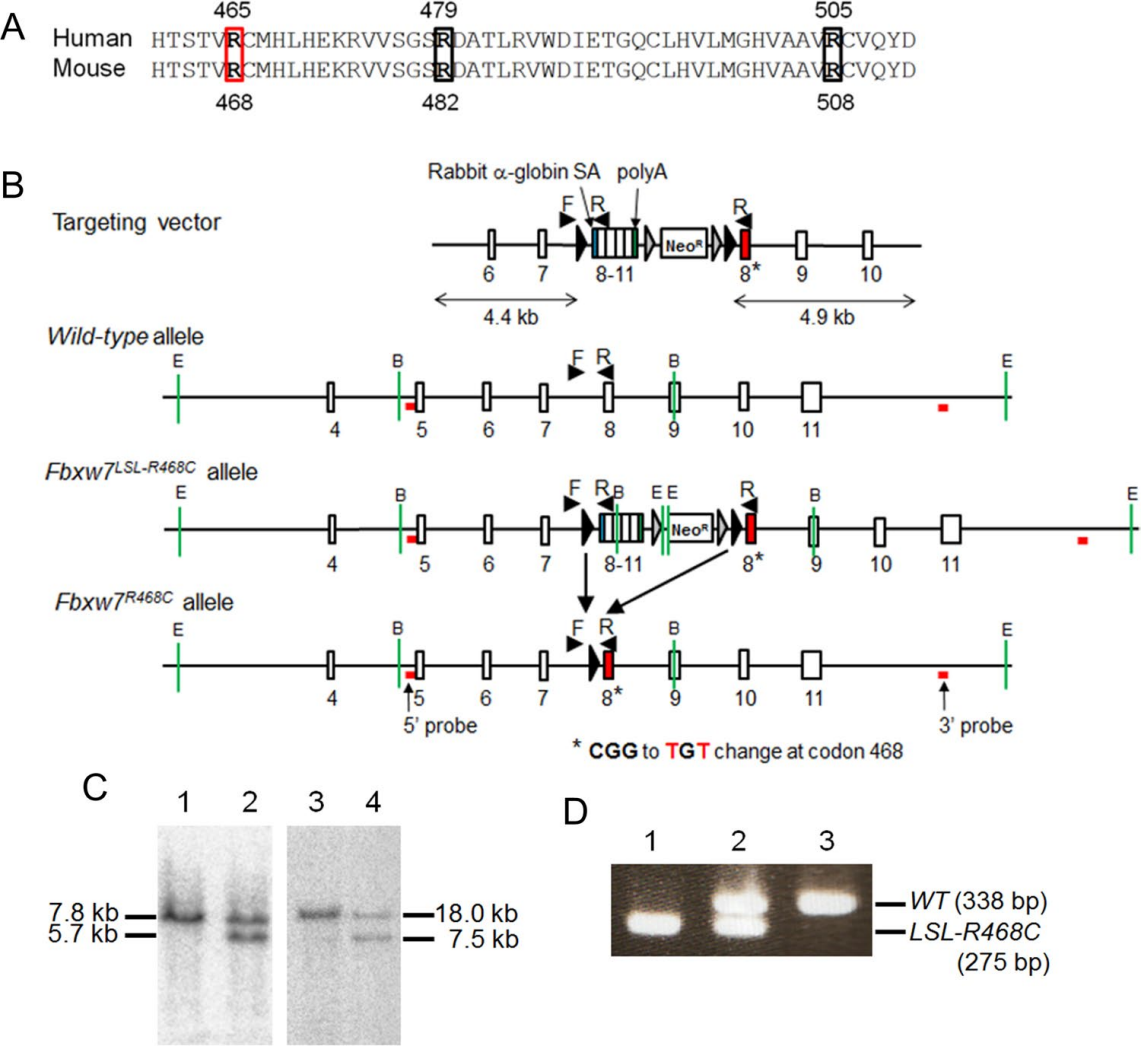
Establishment of conditional *Fbxw7*^*R468C*^ knockin mice. (**A**) Amino acid sequence alignment of human FBXW7 and mouse Fbxw7 proteins. The three mutation hotspots in human primary tumours (R465, R479, and R505) are indicated by boxes. The box in red indicates the arginine residue that was changed to cysteine (R465C in human and R468C in mouse) in this study. (**B**) Schematic illustration of the targeting vector, and the wild-type *Fbxw7*, *Fbxw7*^*LSL-R468C*^, and *Fbxw7*^*R468C*^ alleles. The targeting vector contained the left (4.4 kb) and right (4.9 kb) arms consisting of exons 1–7 and exons 8–11, respectively, and a minigene comprising exons 8–11 of the mouse *Fbxw7* and *Neo*^*R*^ genes flanked by two *FRT* sites (grey triangles) in two *loxP* sites (black triangles) between the left and right arms. Exon 8 (closed red box in the right arm) harboured the mutations C1402T and G1404T (*). Cre-mediated recombination of the *loxP* sites resulted in the deletion of the minigene and expression of the Fbxw7^R468C^ mutant. The locations of genotyping primers (Fbxw7-RC-F and RC-R, denoted by arrowheads with F and R, respectively) and Southern blot probes are indicated. E: *Eco*RI sites, B: *Bam*HI sites. (**C**) Southern blot analysis with probes flanking the 5′ (left panel) and 3′ (right panel) ends of the targeting vector was used to confirm homologous recombination in the *Fbxw7* gene. DNA from embryonic stem (ES) cells carrying *Fbxw7*^+*/*+^ (lanes 1 and 3) and *Fbxw7*^*LSL-R468C/*+^ (lanes 2 and 4) was digested with *Bam*HI (left panel) or *Eco*RI (right panel) and subjected to Southern blot analysis. (D) PCR genotyping of tail DNA from *Fbxw7*^*LSL-R468C/LSL-R468C*^ (lane 1), *Fbxw7*^*LSL-R468C/*+^ (lane 2), and *Fbxw7*^+*/*+^ (lane 3) mice. The wild-type *Fbxw7* allele (*WT*) and the *Fbxw7*^*LSL-R468C*^ knockin allele (*LSL-R468C*) were detected as a band of 338 and 275 bp, respectively.

*FBXW7* is located on chromosome 4q32, where deletions are frequently observed in human cancers. Analysis of the gene has revealed pathogenic mutations in approximately 6% of tumours. Frequent mutations have been reported in cholangiocarcinoma (35%), T-cell acute lymphocytic leukaemia (31%), and colon cancer (9%)^15^. Most of the mutations are missense mutations affecting arginine residues (Arg^465^, Arg^479^, and Arg^505^) within the WD40 domain, which is responsible for substrate recognition (Fig. 1A)^16^, and R465C is the mutation most frequently detected in human primary tumours (Catalogue of Somatic Mutations in Cancer; http://www.sanger.ac.uk/cosmic)^16^.

A mouse line of *Fbxw7*^*R482Q*^ corresponding to the *FBXW7*^*R479Q*^ mutation in humans was previously established by Davis *et al*.^17^. This study reported that mice heterozygous for this mutation died perinatally due to a defect in lung development and other developmental abnormalities, such as eyelid fusion defect and cleft palate. In addition, an *Fbxw7*^*R468C*^ mouse line was previously generated and hematopoiesis was studied^18^. In this model, the *Fbxw7*^*R468C*^ mutation is silenced by introduction of the neomycin-resistant gene cassette in the absence of Cre recombinase^18^. Notably, it was reported that homozygous deletion of *Fbxw7* caused embryonic lethality at E10.5–11.5 due to vascular abnormalities^19,20^.

In this study, we established a novel mouse line carrying a conditional knockin allele of *Fbxw7*^*R468C*^ corresponding to *FBXW*^*R465C*^ in humans. Although similar conditional knockin mouse lines with this mutation have been established and analysed by King *et al*.^18^, the effect of this mutation on mouse development has not been fully elucidated. We therefore generated mice with endogenous expression of *Fbxw7*^*R468C*^, as well as mice whose liver tissues expressed heterozygous and homozygous *Fbxw7*^*R468C*^ alleles, and analysed their phenotypes.

## Results and Discussion

### Generation of transgenic mice containing a conditional knockin allele of the *Fbxw7*^*R468C*^ mutation

We used the Cre-*LoxP* system to generate transgenic mice containing a conditional knockin allele that expresses *Fbxw7*^*R468C*^ only after Cre-mediated recombination. The targeting vector contained a left arm spanning exons 6–7 and a right arm spanning exons 8–10, which harboured two nucleotide changes, C1402T and G1404T (CGG to TGT), at codon 468 in exon 8 to express the *Fbxw7*^*R468C*^ mutation (Fig. 1B). The arms were separated from each other by two *LoxP* sequences. Between these *LoxP* sites, we introduced a minigene cDNA encoding wild-type exons 8–11 preceded by a rabbit α-globin splice accepter site, followed by a polyadenylation *STOP* sequence and the neomycin-resistant cassette (*neo*^*R*^). Consequently, in the absence of Cre recombinase, the targeting vector expressed wild-type *Fbxw7*, including exons 8–11, in the minigene. The introduction of Cre recombinase induced deletion of the *LoxP*-*STOP*-*LoxP* (*LSL*) cassette and expressed *Fbxw7*^*R468C*^ (Fig. 1B).

The vector was introduced into embryonic stem (ES) cells, and homologous recombined clones were identified using Southern blot analysis (Fig. 1C). Mice were generated from the positive clones, and a breeding colony was established in a C57BL/6 background. Genotyping by polymerase chain reaction (PCR) confirmed the inheritance of the *Fbxw7*^*LSL-R468C*^ allele using DNA extracted from mouse tails (Fig. 1D). Although homozygous deletion of the *Fbxw7* gene was reportedly lethal at E10.5–11.5^19,20^, the homozygous mutant mice (*Fbxw7*^*LSL-R468C/LSL-R468C*^) were viable and fertile (Fig. 1D and S1) by the expression of wild-type *Fbxw7* cDNA in the absence of Cre. No apparent developmental abnormalities were observed in either homozygous or heterozygous *Fbxw7*^*LSL-R468C*^ mice.

### Expression of endogenous Fbxw7^R468C^ in embryogenesis

**Figure 2:**
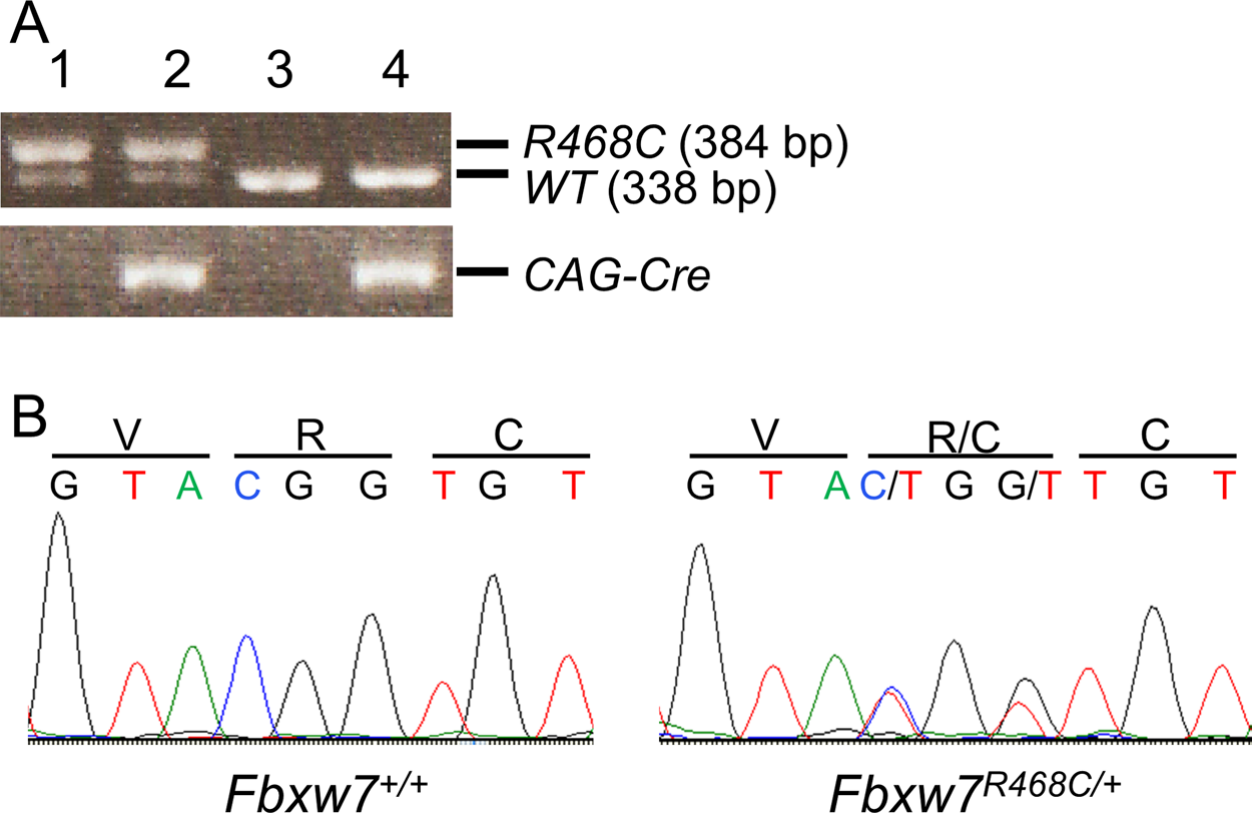
Cre-mediated gene recombination and expression of *Fbxw7*^*R468C*^ transcripts. (**A**) PCR genotyping of DNA from E18.5 embryos. A male *Fbxw7*^*LSL-R468C/*+^ mouse was crossed with a female *CAG-Cre* mouse. The mutant *Fbxw7*^*R468C*^ allele (*R468C*) corresponded to a band of 384 bp and the wild-type allele (*WT*) to a band of 338 bp. Lane 1: *Fbxw7*^*R468C/*+^, lane 2: *Fbxw7*^*R468C/*+^;*CAG-Cre*, lane 3: *Fbxw7*^+*/*+^, and lane 4: *Fbxw7*^+*/*+^;*CAG-Cre*. (**B**) PCR direct sequence analysis of RT-PCR products from *Fbxw7*^+*/*+^ and *Fbxw7*^*R468C/*+^ mice. RNA was extracted from mouse embryonic fibroblasts (MEFs) isolated from E12.5 *Fbxw7*^+*/*+^ and *Fbxw7*^*R468C/*+^ embryos.

To investigate the effect of endogenous expression of the Fbxw7^R468C^ mutant on mouse development, we next generated mice that permanently express Fbxw7^R468C^ in all tissues. We used *CAG-Cre* mice that ubiquitously express Cre recombinase^21^. In progeny mice from heterozygous female *CAG*-*Cre* parents that possess a maternal effect, the target transgenes undergo complete excision of the floxed sequence in the embryos, even in the absence of *CAG-Cre* inheritance^21^. Thus, we crossed male *Fbxw7*^*LSL-R468C/*+^ mice to female *CAG-Cre* mice. Genotyping of the surviving offspring revealed that no living newborns but some dead offspring harboured the *Fbxw7*^*R468C*^ allele. Additional genotyping of E18.5 embryos detected the successful recombination of the *Fbxw7*^*R468C*^ allele irrespective of the inheritance of the *CAG-Cre* transgene, with an expected Mendelian ratio of 1:1 at E18.5 (Fig. 2A). Furthermore, *Fbxw7*^*R468C*^ transcripts were confirmed in the *Fbxw7*^*R468C/*+^ embryos (Fig. 2B). At this time point, there were no obvious differences in the gross appearance of *Fbxw7*^*R468C/*+^ and wild-type embryos. Therefore, we concluded that the *Fbxw7*^*R468C/*+^ embryos died around birth. The results are consistent with the phenotype of the *Fbxw7*^*R482Q/+*^ mutant embryos reported by Davis *et al*.^17^.

### Developmental defects in *Fbxw7*^*R468C/*+^ mice

**Figure 3:**
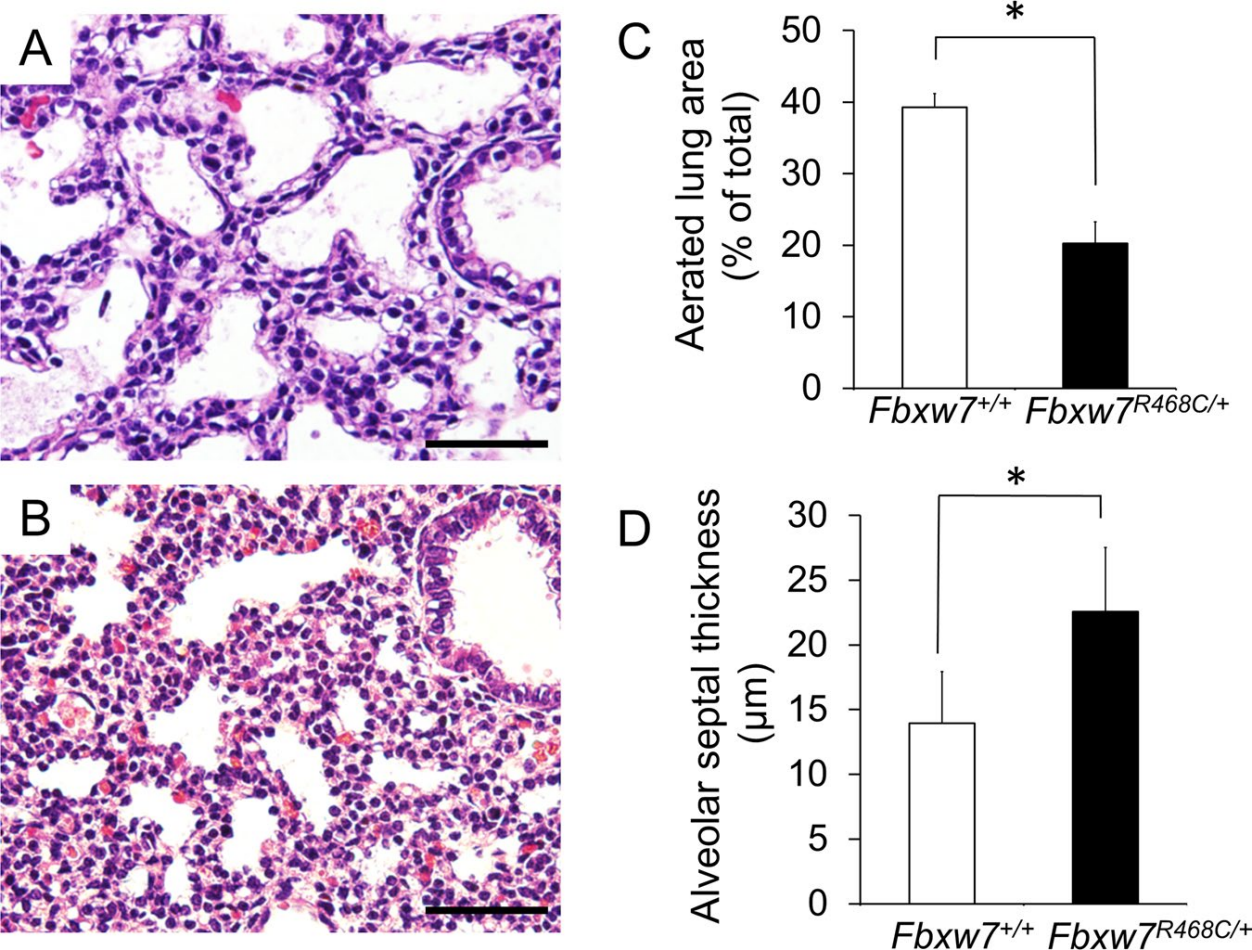
Histological analysis of lungs in wild-type and *Fbxw7*^*R468C/*+^ mice. (**A**,**B**) Representative hematoxylin and eosin (H&E) staining of the lungs of wild-type (**A**) and *Fbxw7*^*R468C/*+^ (**B**) mice at E18.5. Bar, 50 μm. (**C**) Aerated lung area in wild-type and *Fbxw7*^*R468C/*+^ mice at E18.5. (**D**) Thickness of the alveolar septum in wild-type and *Fbxw7*^*R468C/*+^ mice at E18.5. Error bars indicate the mean ± standard deviation (SD). *P < 0.05.

**Figure 4:**
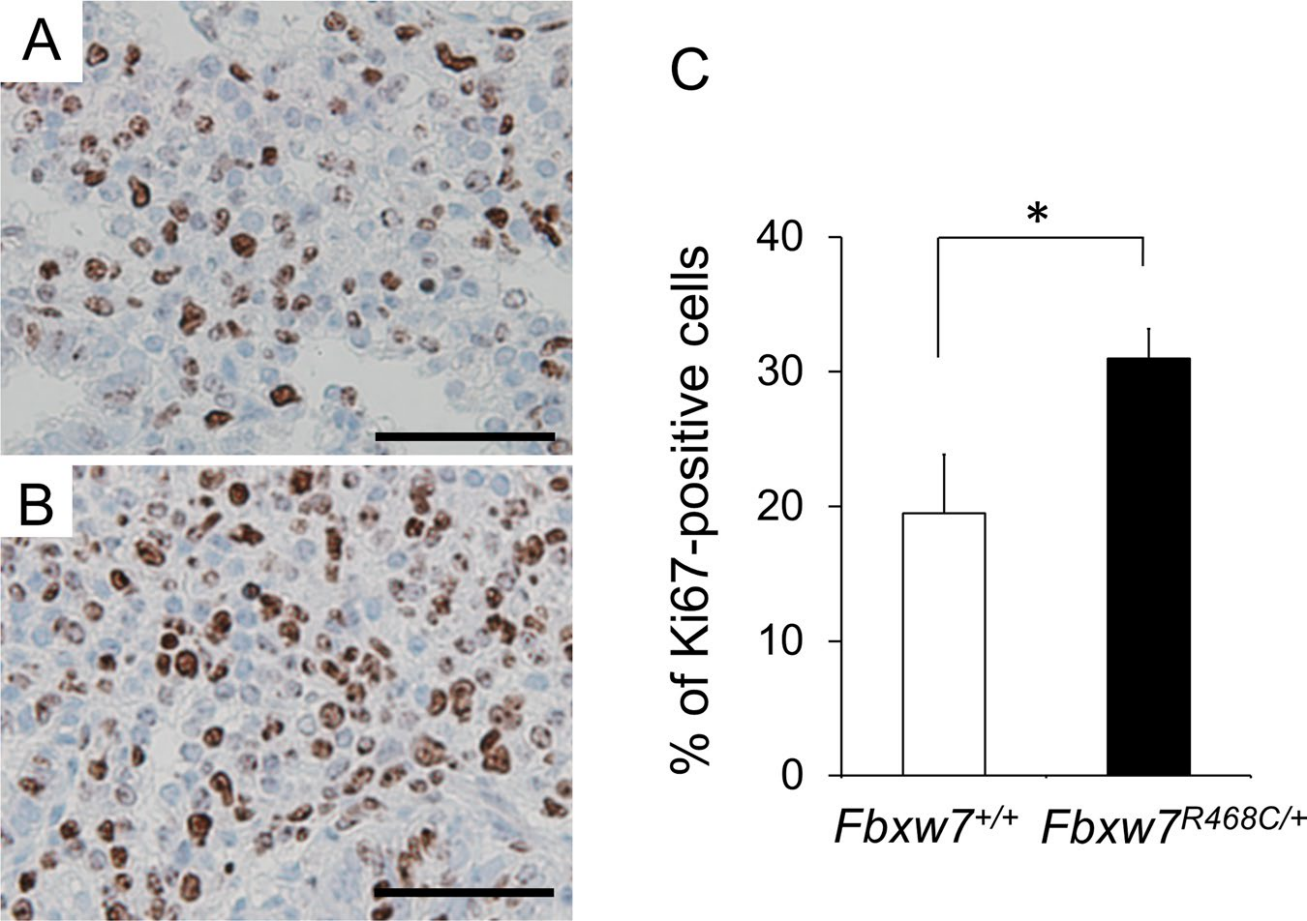
Cell proliferation in the lungs of wild-type and *Fbxw7*^*R468C/*+^ mice. (**A**,**B**) Immunohistochemical staining of Ki67 in the lungs of wild-type (**A**) and *Fbxw7*^*R468C/*+^ (**B**) mice. Bar, 50 μm. (C) Quantification of Ki67-positive cells in wild-type and *Fbxw*^*R468C/*+^ mutant mice. Error bars indicate the mean ± SD. *P < 0.05.

To elucidate the cause of perinatal lethality of *Fbxw7*^*R468C/*+^ mice, we performed histological analysis of E18.5 embryos in both *Fbxw7*^*R468C/*+^ and wild-type mice. We first focused on the lung in *Fbxw7*^*R468C/*+^ mice because defects in lung development have been reported in the *Fbxw7*^*R482Q/+*^ mice^17^. There were apparent differences in the lung histology of wild-type and mutant mice (Fig. 3A and B). Aerated lung areas in *Fbxw7*^*R468C/*+^ mice were small and their alveolar septa were thickened compared with septa in wild-type littermates (Fig. 3A–D), suggesting that respiratory failure is the cause of perinatal lethality in *Fbxw7*^*R468C/*+^ mice. To clarify the cause of the lung abnormalities in E18.5 *Fbxw7*^*R468C/*+^ mice, cell proliferation was examined by immunohistochemical staining of Ki67 in the lung. We observed a significant increase in the number of Ki67-positive cells in the *Fbxw7*^*R468C/*+^ lung compared with the wild-type lung (Fig. 4A–C), which suggests that enhanced cell proliferation caused the thickening of the alveolar septa in the *Fbxw7*^*R468C/*+^ lung at E18.5.

We also investigated other developmental defects, including eyes-open at birth (EOB) and cleft palate, in our *Fbxw7*^*R468C/*+^ mice model, since these defects were also previously reported in *Fbxw7*^*R482Q/*+^ mice^17^. EOB and cleft palate were consistently observed in 17 of 102 (17%) (Figure S2A–D) and one of 78 (1.3%) (Figure S2E and F) mice, respectively. However, the frequencies of these two abnormalities in *Fbxw7*^*R468C/*+^ mice were lower than those reported in *Fbxw7*^*R482Q/*+^ mice^17^. Although the difference in frequencies of the abnormalities may only reflect the difference in genetic background or housing conditions, it is possible that the functions of the R468C and R482Q mutants are not completely identical.

Since lung, eye, and palate abnormalities were not reported in *Fbxw7*^+*/–*^ mice, the defects of these organs in *Fbxw7*^*R468C/*+^ mice, as well as *Fbxw7*^*R482Q/*+^ mice, are not due to the haploinsufficiency of *Fbxw7*, but rather due to its function as the ‘just enough’ or ‘dominant negative’ tumour suppressor proposed by Davis *et al*.^16^.

### Effect of the *Fbxw7*^*R468C/*+^ mutation on substrate stability

**Figure 5:**
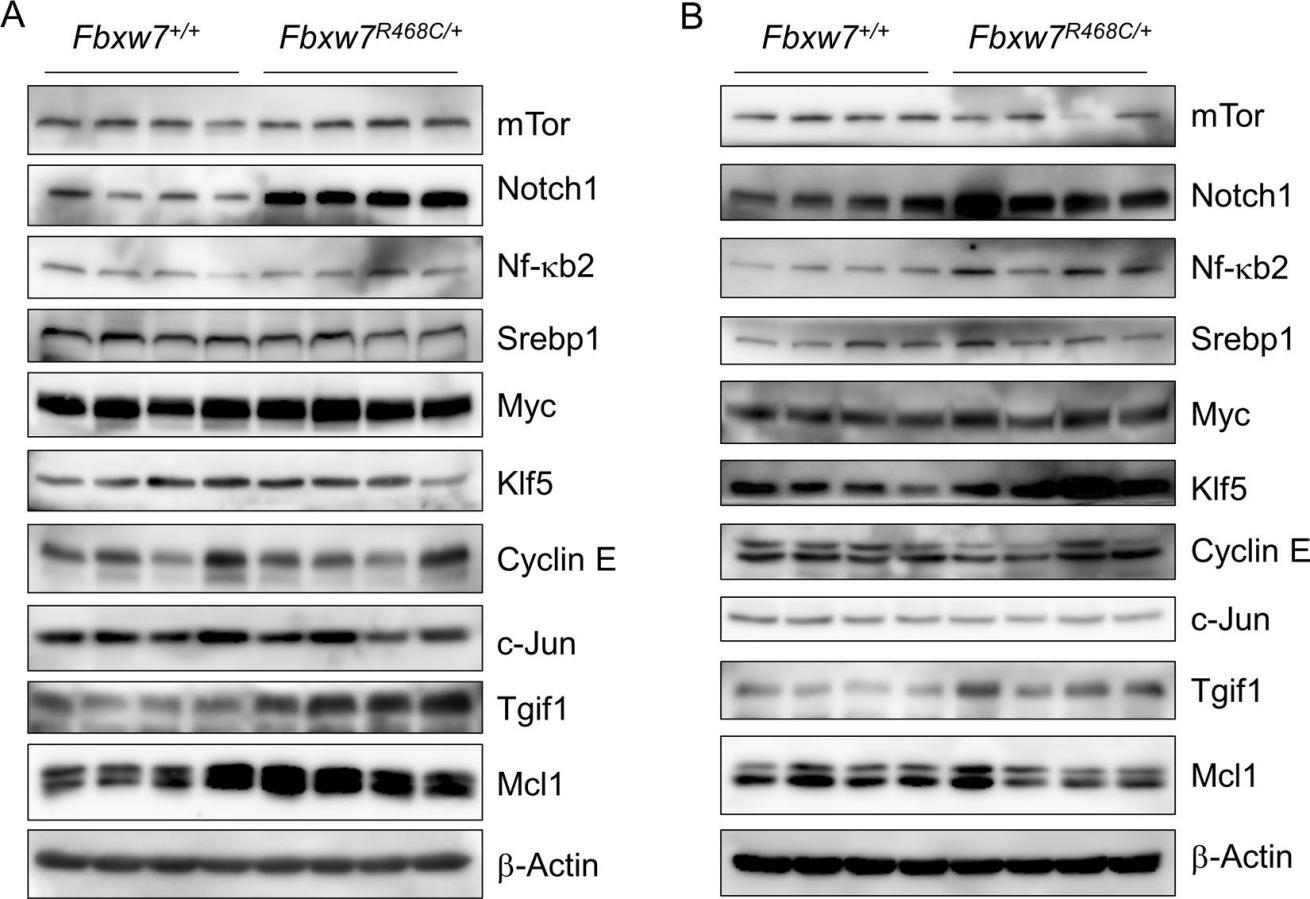
Expression of Fbxw7 substrates in wild-type and mutant embryos and MEFs. (**A**) Expression of Fbxw7 substrates in wild-type and heterozygous mouse embryonic heads. The lysates from E12.5 *Fbxw7*^+*/*+^ and *Fbxw7*^*R468C/*+^ embryos (n = 4 each) were analysed by immunoblot analysis. (**B**) Expression of Fbxw7 substrates in wild-type and heterozygous MEFs. The lysates from MEFs with each genotype (*Fbxw7*^+*/*+^ and *Fbxw7*^*R468C/*+^; n = 4 each) were analysed as described in (**A**).

Recent studies of FBXW7 have resulted in an increased number of its substrate proteins, including mTOR, NOTCH1, SREBP1, NF-κB2, MYC, KLF5, Cyclin E, c-JUN, MCL1, and TGIF1^22,23^. To investigate the effect of the *Fbxw7*^*R468C*^ mutation on substrate stability, we analysed their expression levels using lysates of embryos and mouse embryonic fibroblasts (MEFs) from wild-type and *Fbxw7*^*R468C/*+^ mice by immunoblotting (Fig. 5A and B). Although the expression levels of Notch1, Tgif1, and Mcl1 were elevated in the lysates of *Fbxw7*^*R468C/*+^ embryos compared with those of wild-type embryos, no differences were observed in the levels of mTor, NF-κB2, Srebp1, Myc, Klf5, Cyclin E, and c-Jun (Fig. 5A). Interestingly, the expression levels of Notch1, NF-κB2, Klf5, and Tgif1 were increased, but those of mTor, Srebp1, Myc, Cyclin E, c-Jun, and Mcl1 were unchanged in the lysates of MEFs from *Fbxw7*^*R468C/*+^ mice compared with wild-type mice (Fig. 5A and B). In addition, real-time PCR analyses revealed no difference in the mRNA expression levels of some Fbxw7 substrates between MEF cells from *Fbxw7*^*R468C/*+^ mice and those from wild-type mice (Figure S3). These data suggest that the R468C mutation affects the stability of specific target proteins in a cell type-specific manner. In accordance with our observation, it has been reported that the *Fbxw7*^*R482Q*^ mutation affects some of the reported Fbxw7 target proteins in a cell type-specific manner^17,24^.

### The effect of liver-specific *Fbxw7*^*R468C*^ knockin on biliary abnormalities and tumourigenesis

**Figure 6:**
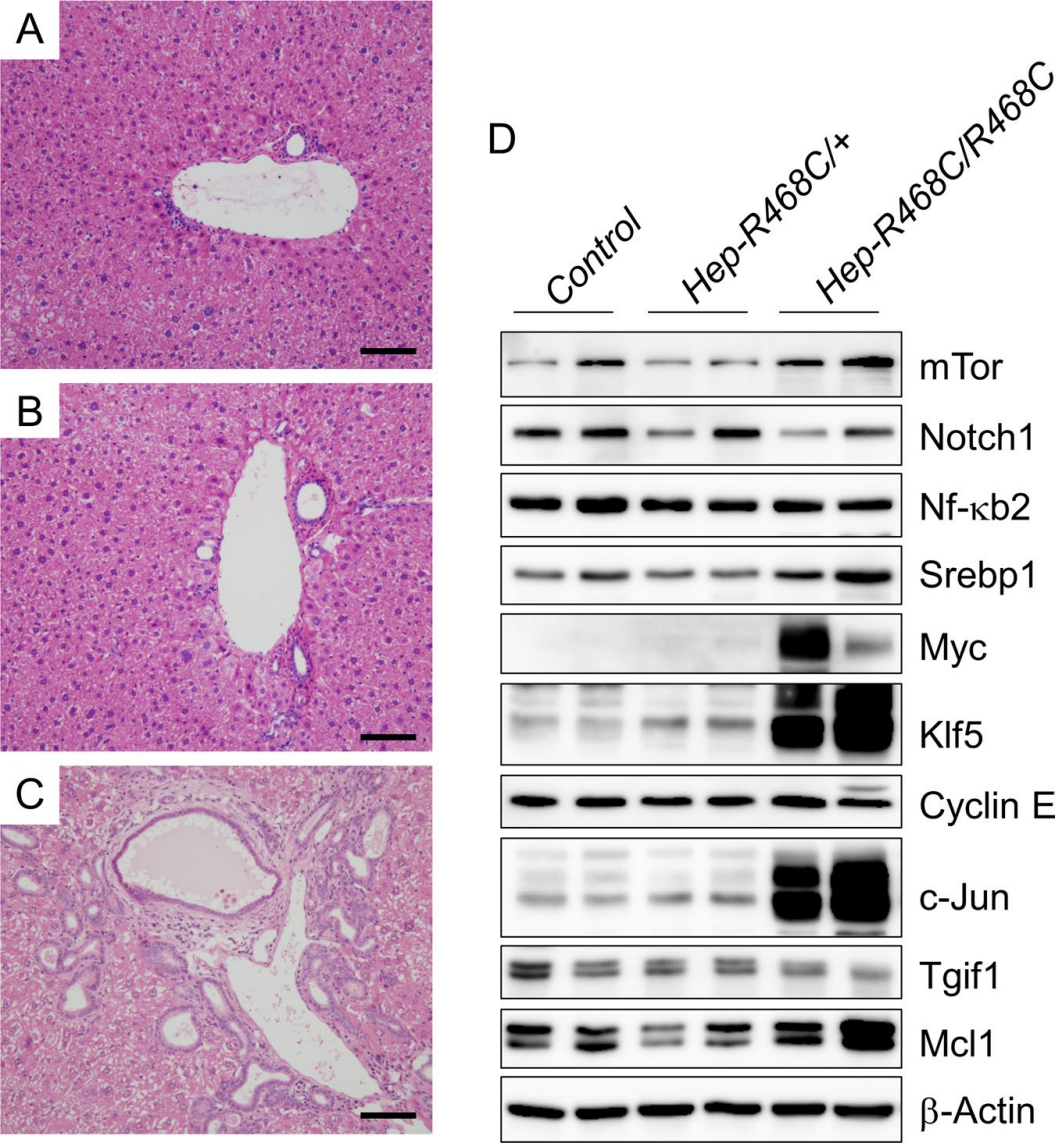
Analysis of livers in mice with heterozygous and homozygous *Fbxw*^*R468C*^ mutations. (**A**–**C**) Representative H&E staining of the livers of control *Alb-Cre* (^+^/^+^) (**A**), *Alb-Cre*;*Fbxw7*^*LSL-R468C/*+^ (*Fbxw7*^*Hep-R468C/*+^;*Hep-R468C/*^+^) (**B**), and *Alb-Cre*;*Fbxw7*^*LSL-R468C/LSL-R468C*^ (*Fbxw7*^*Hep-R468C/Hep-R468C*^;*Hep-R468C/Hep-R468C*) (**C**) mice at 12 months of age. Bar, 100 μm. (**D**) Expression of Fbxw7 substrates in the livers of mice with the indicated genotype.

*FBXW7* mutations are involved in various malignancies, including intrahepatic cholangiocarcinoma^15,25^. In addition, homozygous deletion of *Fbxw7* in the murine liver reportedly induced hyperplasia of the bile duct and biliary hamartomas, also known as von Meyenburg complexes^26^. Therefore, we next investigated the livers of conditional *Fbxw7*^*R468C*^ knockin mice. We crossed *Fbxw7*^*LSL-R468C/*+^ mice with *Alb-Cre* mice, and generated *Alb-Cre*;*Fbxw7*^*LSL-R468C/*+^ (*Fbxw7*^*Hep-R468C/*+^) and *Alb-Cre*;*Fbxw7*^*LSL-R468C/LSL-R468C*^ (*Fbxw7*^*Hep-R468C/R468C*^) mice, whose liver tissues expressed the heterozygous and homozygous *Fbxw7*^*R468C*^ allele, respectively. These mice were alive and did not show obvious developmental defects. There were no apparent abnormalities in the livers of *Fbxw7*^*Hep-R468C/*+^ mice until 12 months of age (Fig. 6A and B). In contrast, dilatation and hyperplasia of bile ducts were observed throughout the livers of *Fbxw7*^*Hep-R468C/R468C*^ mice at the age of 12 months (Fig. 6C). Consistent with the report by Onoyama *et al*., our data imply that wild-type Fbxw7 protein is necessary for the development of a normal bile duct.

We further assessed the expression of Fbxw7 target proteins in the livers of control (*Alb-Cre*), heterozygous (*Fbxw7*^*Hep-R468C/*+^), and homozygous (*Fbxw7*^*Hep-R468C/R468C*^) knockin mice by immunoblot analysis. We did not observe a significant difference in the expression of target proteins between heterozygous (*Fbxw7*^*Hep-R468C/*+^) and control mice (Fig. 6D); however, the expression of Myc, Klf5, and c-Jun was markedly induced in the livers of homozygous mice (*Fbxw7*^*Hep-R468C/R468C*^). In addition, the expression of mTor, Srebp1, and Mcl1 was modestly increased in the livers of homozygous mice (Fig. 6D). These data suggest that the accumulation of these proteins may be involved in the abnormal development of the bile duct in *Fbxw7*^*Hep-R468C/R468C*^ mice.

**Figure 7:**
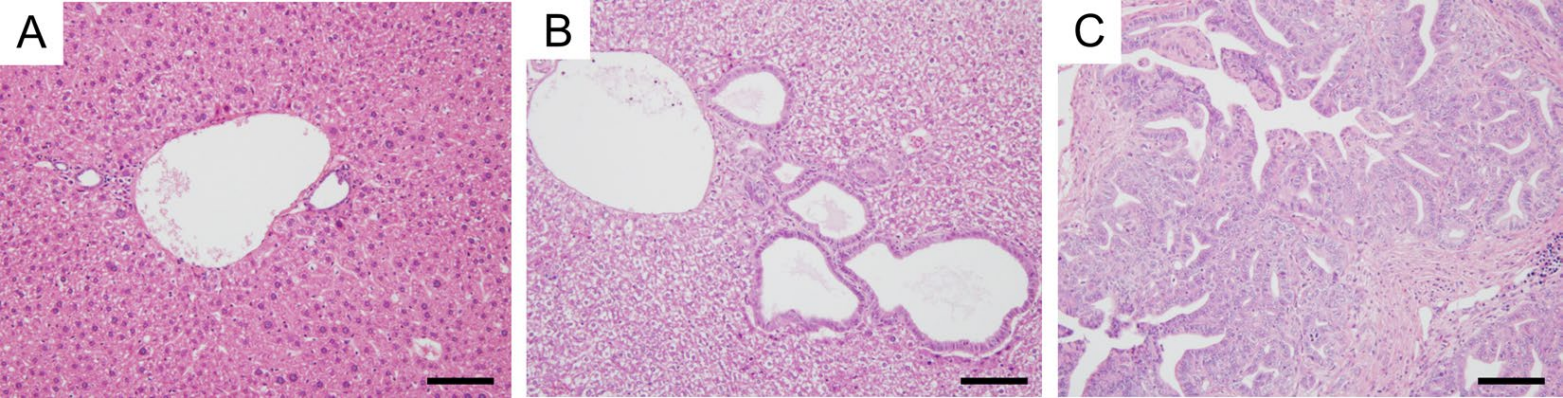
Liver histology in mice with heterozygous and homozygous *Fbxw*^*R468C*^ mutations in combination with an oncogenic *Kras*^*G12D*^ mutation. (**A**–**C**) Representative H&E staining of the livers of *Alb-Cre*;*Kras*^*LSL-G12D/*+^ (**A**) and *Alb-Cre*; *Kras*^*LSL-G12D/*+^;*Fbxw7*^*LSL-R468C/*+^ mice at the age of 8 months (**B**) and *Alb-Cre*;*Kras*^*LSL-G12D/*+^;*Fbxw7*^*LSL-R468C/LSL-R468C*^ mice at the age of 8 weeks (**C**). Bar, 100 μm.

To further investigate the role of the *Fbxw7*^*R468C*^ mutation in murine liver tumourigenesis, we introduced a liver-specific *Fbxw7*^*R468C*^ mutation in combination with an oncogenic *Kras* mutation. Mice carrying the conditional knockin allele of the *Kras*^*G12D*^ mutation (*Kras*^*LSL-G12D/*+^) were crossed with *Alb-Cre* mice and *Fbxw7*^*LSL-R468C/*+^ mice to generate *Alb-Cre*;*Kras*^*LSL-G12D/*+^;*Fbxw7*^*LSL-R468C/*+^ (*Kras*^*Hep-G12D/*+^;*Fbxw7*^*Hep-R468C/*+^) and *Alb-Cre*;*Kras*^*LSL-G12D/*+^;*Fbxw7*^*LSL-R468C/LSL-R468C*^ (*Kras*^*Hep-G12D/*+^;*Fbxw7*^*Hep-R468C/R468C*^) mice. Although no abnormal histological findings were detected in *Alb-Cre*;*Kras*^*LSL-G12D/*+^ (*Kras*^*Hep-G12D/*+^) mice, carrying the liver-specific *Kras*^*G12D*^ alone, until 8 months after birth, the heterozygous *Fbxw7*^*LSL-R468C*^ mutation in combination with the oncogenic *Kras* mutation induced bile duct dilation and hyperplasia in some mice at the age of 8 months (Fig. 7A and B). Furthermore, the homozygous *Fbxw7*^*LSL-R468C*^ mutation in combination with oncogenic *Kras* induced cholangiocarcinoma-like lesions composed of dysplastic dust-like structures surrounded by fibrosis in all mice within 2 months of birth (Fig. 7C). These findings suggest the effects of the *Fbxw7*^*R468C*^ mutation in the promotion of cholangiocarcinogenesis. Considering that the majority of *FBXW7* mutations in human cancer are heterozygous and a previous study revealed the promotive effects of intestinal polyps by the heterozygous *Fbxw7*^*R482Q*^ mutation on an *Apc* mutant background^24^, further studies are required to follow up the phenotypes of the liver-specific heterozygous *Fbxw7*^*R468C*^ mutant mice with or without oncogenic *Kras* mutation.

Collectively, we successfully established a novel mouse line that conditionally and endogenously expresses the *Fbxw7*^*R468C*^ mutant, the mouse counterpart of human *FBXW7*^*R465C*^. Mutant mice showed several developmental abnormalities, including lung defects, EOB, and cleft palate. Furthermore, endogenous expression of the *Fbxw7*^*R468C*^ mutant affected a subset of reported target proteins, such as Notch1 and Tgif1, in a tissue-dependent manner. In addition, homozygous expression of mutant *Fbxw7*^*R468C*^ induced dilatation of the bile duct in the murine liver, which was accompanied by the remarkable augmentation of Myc, Klf5, and c-Jun proteins. Furthermore, homozygous expression of this mutant cooperated with oncogenic *Kras* to induce intrahepatic cholangiocarcinoma-like lesions. This conditional knockin mouse line of the cancer-associated hotspot mutation of *FBXW7* should therefore be a useful tool for elucidating the mechanisms underlying human neoplasms that are associated with *FBXW7* mutation.

## Materials and Methods

### Generation of the conditional *Fbxw7*^*R468C*^ allele

Conditional *Fbxw7*^*R468C*^ knockin mice were generated by gene targeting. The targeting vector was constructed by cloning of the left, middle, and right arms in the pEZ-Frt-loxP-DT vector. Both fragments of the left (4.4 kb) arm containing exons 1–7, and the right (4.9 kb) arm containing exons 8–11 were amplified by PCR using bacterial artificial clone RP23-269G19 as a template. Two mutations (C1402T and G1404T) were introduced into exon 8 on the right arm using site-directed mutagenesis. Additionally, a fragment of cDNA consisting of exons 8–11 of mouse *Fbxw7* was amplified using a gene-specific primer set containing a rabbit α-globin splice acceptor at the 5′ end and a polyadenylation signal at the 3′ end with wild-type *Fbxw7* cDNA as a template. The PCR product was subsequently cloned into two *loxP* sites (black triangles) of the pEZ-Frt-loxP-DT vector using the In-Fusion HD Cloning System (TAKARA) according to the manufacturer’s instructions. The minigene containing exons 8–11 of *Fbxw7* cDNA and the *Neo*^*R*^ gene flanked by two *FRT* sites (grey triangles) in the LSL cassette were cloned between the left and right arms of the targeting vector. The construct was electroporated into EGR-101 ES cells^27^ and G418-resistant clones were selected. Homologous recombinant ES clones were screened by Southern blotting using the probes as indicated in Fig. 1B. Positive clones were injected into mouse C57BL/6 blastocysts and chimeric mice were generated by an aggregation method^28^. Chimeric male mice were bred to female mice to achieve germline transmission.

To generate mice carrying the *Fbxw7*^*R468C*^ mutation in the whole body, male *Fbxw7*^*LSL-R468C/*+^ mice were crossed with female *CAG-Cre* mice (RIKEN BRC), in which Cre recombinase is expressed ubiquitously under the control of the *CAG* promoter^21^. To generate liver-specific *Fbxw7*^*R468C*^ knockin mice, *Fbxw7*^*LSL-R468C/*+^ mice were crossed with the *Alb-Cre* mice (Jackson Laboratory)^29^. To generate mice carrying liver-specific both *Fbxw7*^*R468C*^ and *Kras*^*G12D*^, the *Fbxw7*^*LSL-R468C/*+^ mice were crossed with the conditional *Kras*^*G12D*^ knockin mice (*Kras*^*LSL-G12D*^) (Jackson Laboratory)^30^ and the *Alb-Cre* mice. All mice were bred on a C57BL/6 genetic background. All mice were bred in a C57BL/6 genetic background.

Mice were housed in specific pathogen-free conditions within the animal care facility at the Institute of Medical Science, the University of Tokyo. All of the experimental protocols were approved by the Animal Care and Use Committee of the University of Tokyo and conducted in accordance with the Guidelines for the Care and Use of Laboratory Animals of the University of Tokyo (approval nos. PA11-03 and PA16-41).

### Genotyping

Genomic DNA from tail tips was extracted according to the standard phenol extraction/purification procedure. Genotyping was carried out using the following PCR primers: Fbxw7-RC-F (5′-CAACCAGTGCTCTAAATCACT-3′) and Fbxw7-RC-R (5′-CTCCACCTGTATGTCCCACTA-3′) for *Fbxw7* alleles (Fig. 1B) and Cre-F (5′-GCATTACCGGTCGATGCAACGAGTGATGAG-3′) and Cre-R (5′-GAGTGAACGAACCTGGTCGAAATCAGTGCG-3′) for the *Cre* transgene. The wild-type allele was identified as a 338 bp PCR product, and the *Fbxw7*^*LSL-R468C/*+^ knockin allele was identified as a 275 bp product. After Cre-mediated recombination, the *Fbxw7*^*R468C*^ knockin allele was identified as a 384 bp PCR product. The *Cre* transgene was detected as a PCR product of 408 bp. All PCRs were performed using GoTaq G2 Hot Start DNA polymerase (Promega) with 33 cycles at 98 °C for 30 sec, 60 °C for 30 sec, and 72 °C for 1 min.

### Detection of the *Fbxw7*^*R468C*^ mutation

For analysis of the *Fbxw7* mutations in genomic DNA, a region encompassing codon 468 was amplified using DNA from the tail tips and the following set of primers: Fbxw7-D/R-F (5′-GTCATCACAGATGAGAGACA-3′) and Fbxw7-D-R (5′-CTATGAATCCCAGAGTCACA-3′). For analysis of the *Fbxw7* mutations in mRNA, another region encompassing the codon was amplified using cDNA from MEFs and the following set of primers: Fbxw7-D/R-F and Fbxw7-R-R (5′-CATCAGGACGTGTAAACACT-3′). cDNA was synthesised using the Transcriptor First Strand cDNA Synthesis Kit (Roche Diagnostics) with 1 µg of total RNA extracted from MEFs using an RNeasy Mini Kit (Qiagen). PCR reactions were performed using KOD Plus DNA Polymerase (Toyobo) with 35 cycles at 98 °C for 10 sec, 60 °C for 30 sec, and 68 °C for 30 sec. To confirm the sequence, PCR products were purified using a PCR purification kit (Qiagen) and sequenced on an Applied Biosystems 3730xl DNA Analyzer (Thermo Fisher Scientific) with the BigDye Direct Cycle Sequencing Kit (Thermo Fisher Scientific). The Fbxw7-D/R-F primer was used for sequencing.

### Histology and immunohistochemistry

E18.5 embryos were collected, fixed with 10% formalin, and embedded in paraffin for sectioning. Sections (3 μm) were stained with hematoxylin and eosin. For immunohistochemistry, sections were dewaxed in xylene and hydrated by immersion in 100% ethanol and distilled water. Antigen retrieval was performed by boiling for 10 min in 10 mM citrate solution (pH 6.0). Sections were incubated for 10 min in 0.3% H_2_O_2_-methanol for blocking endogenous peroxidase, blocked with 10% serum for 30 min at room temperature, and incubated overnight at 4 °C with anti-Ki67 antibodies (1:50; Clone TEC-3, DAKO). Slides were rinsed in phosphate-buffered saline and peroxidase activity was detected using the Vectastain Elite ABC Kit (Vector Laboratories) and ImmPACT DAB substrate (Vector Laboratories). Nuclei were counterstained with hematoxylin for 1 min.

### Immunoblot analysis

Lysates were extracted from the heads of E12.5 embryos, MEFs, and adult livers in radioimmunoprecipitation assay buffer (50 mM Tris-HCl, pH 8.0, 150 mM NaCl, 0.5% sodium deoxycholate, 1% Nonidet P-40, and 0.1% SDS) supplemented with a Protease Inhibitor Cocktail Set III (Calbiochem). Immunoblot analysis was performed using the following primary antibodies: mTOR (7C10, Cell Signaling), Notch1 (D1E11, Cell Signaling), NF-κB2 (18D10, Cell Signaling), Myc (Y69, Abcam), Klf5 (ab24311, Abcam and G-7, Santa Cruz), c-Jun (H-79 and G-4, Santa Cruz), TGIF (H-172, Santa Cruz), Cyclin E (C19, Delta Biolabs), SREBP1 (2A4, Novus Biologicals and H-160, Santa Cruz), and β-actin (AC-15, Sigma). Each primary antibody was used at a 1:1000 dilution. Horseradish peroxidase-conjugated goat anti-mouse or anti-rabbit IgG (GE Healthcare) was used as the secondary antibody. Immunoreactive proteins were visualised using the SuperSignal West Pico Chemiluminescent Substrate (Thermo Fisher Scientific) according to the manufacturer’s instructions.

### Real-time PCR

Total RNA from MEFs was extracted with Trizol (Invitrogen) according to the manufacturer’s instructions. cDNA was synthesised using the Transcriptor First Strand cDNA Synthesis Kit (Roche Diagnostics). Real-time PCR was performed using the qPCR Kapa SYBR Fast ABI Prism Kit (Kapa Biosystems) on StepOnePlus (Thermo Fisher Scientific). The amount of transcript was determined using the relative standard curve method, and *Gapdh* was used as an internal control. The primer sequences used are shown in Table S1.

### Statistical analysis

Statistical significance between the two groups was determined using Student’s *t*-test. Data are displayed as the mean ± standard deviation. Probability values less than 0.05 were considered statistically significant.

## Electronic supplementary material

Supplementary information
