# Supplementary material for: Establishment and analysis of a novel mouse line carrying a conditional knockin allele of a cancer-specific FBXW7 mutation
Source: Sci Rep. 2018 Jan 31;8:2021. doi: 10.1038/s41598-018-19769-1 (PMC5792591; doi:10.1038/s41598-018-19769-1)
Supplement: Supplementary file 1 — Supplementary information [file 41598_2018_19769_MOESM1_ESM.pdf]

Establishment and analysis of a novel mouse line carrying a conditional knockin allele of cancer-specific *FBXW7* mutation

Tsuneo Ikenoue, Yumi Terakado, Chi Zhu, Xun Liu, Tomoyuki Ohsugi, Daisuke Matsubara, Tomoki Fujii, Shigeru Kakuta, Sachiko Kubo, Takuma Shibata, Kiyoshi Yamaguchi, Yoichiro Iwakura, Yoichi Furukawa

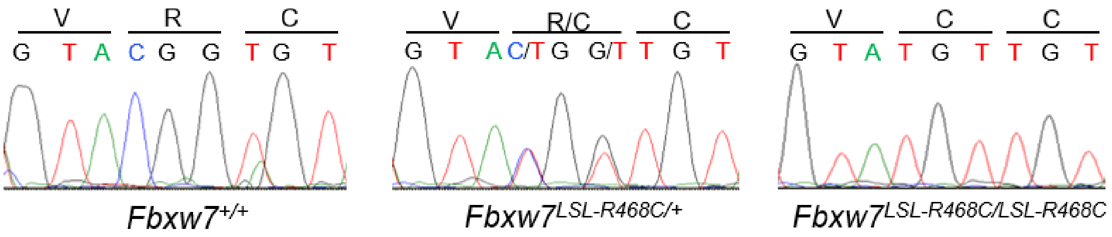

Supplementary Figure S1. Genomic sequences of the region encoding codon 467-469 in *Fbxw7*$^{+/+}$, *Fbxw7*$^{LSL\text{-}R468C/+}$, and *Fbxw7*$^{LSL\text{-}R468C/LSL\text{-}R468C}$ mice.

Heterozygous and homozygous CGG to TGT changes at codon 468 were detected in the DNA extracted from the tails of *Fbxw7*$^{LSL\text{-}R468C/+}$ and *Fbxw7*$^{LSL\text{-}R468C/LSL\text{-}R468C}$ mice, respectively.

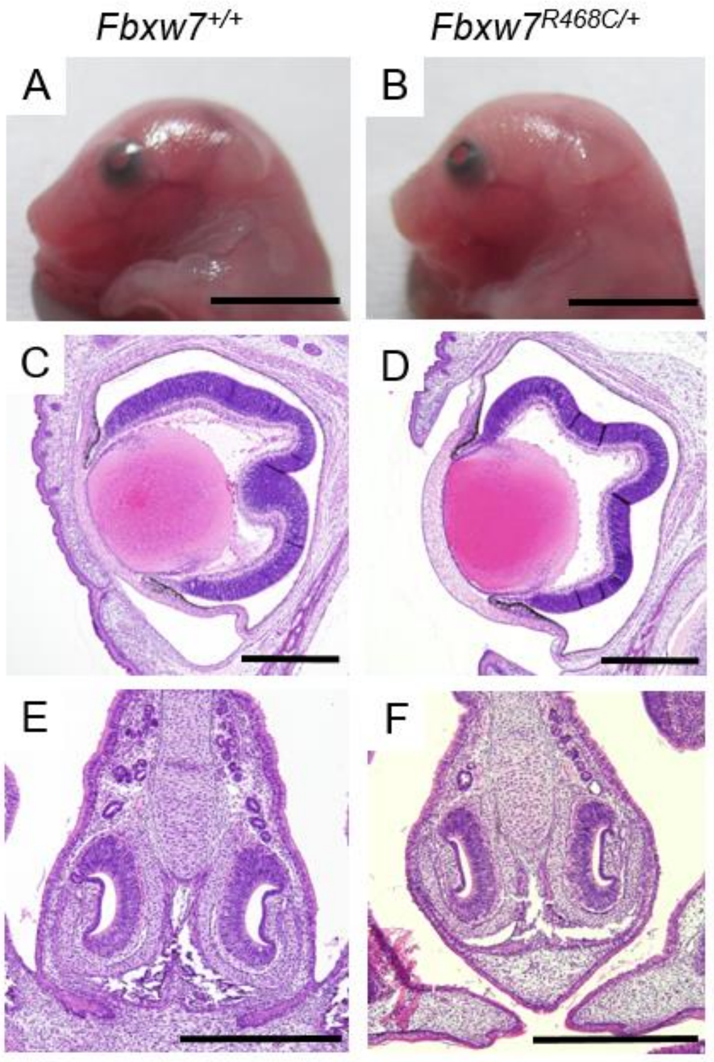

Supplementary Figure S2. Developmental defects of the eye lids and oral cavity in *Fbxw7*$^{R468C/+}$ mice.

(A-D) Gross appearance (A, B) and H&E staining (C, D) of the wild-type (A, C) and *Fbxw7*^{R468C}/+ (B, D) mice. Bar, 5 mm (A, B); 500 μm (C, D). Eyes open at birth (EOB) phenotype observed in *Fbxw7*^{R468C}/+ mice (B and D) but not in wild-type mice (A and C). (E, F) H&E staining of a *Fbxw7*^{R468C}/+ mouse showing cleft palate (F). Normal palate appearance was observed in wild-type mice (E). Bar, 500 μm.

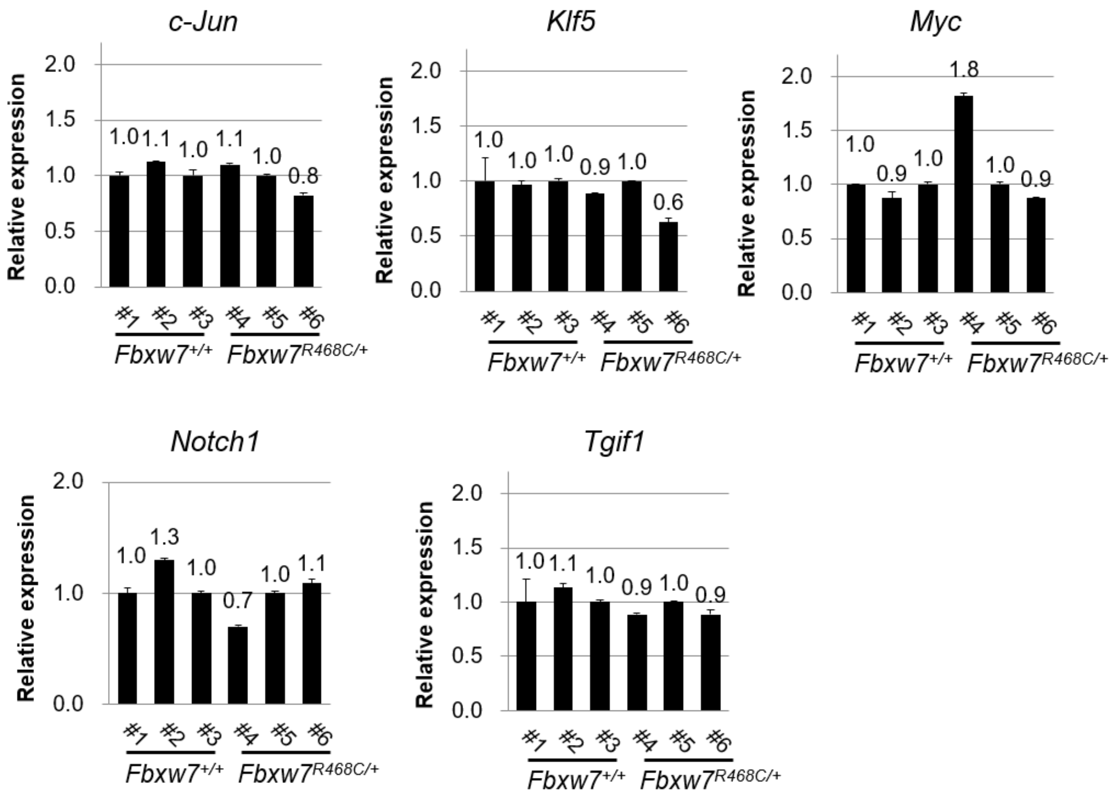

Supplementary Figure S3. mRNA expression of Fbxw7 targets in MEFs from *wild-type* and *Fbxw7*$^{\mathit{R468C/+}}$ embryos.

Expression levels of mRNA of each gene in MEFs from three wild-type (#1-3) and three mutant (#4-6) embryos were determined by qRT-PCR. The relative expression ratio was calculated in comparison to *Gapdh*. Error bars indicate mean ± SD.

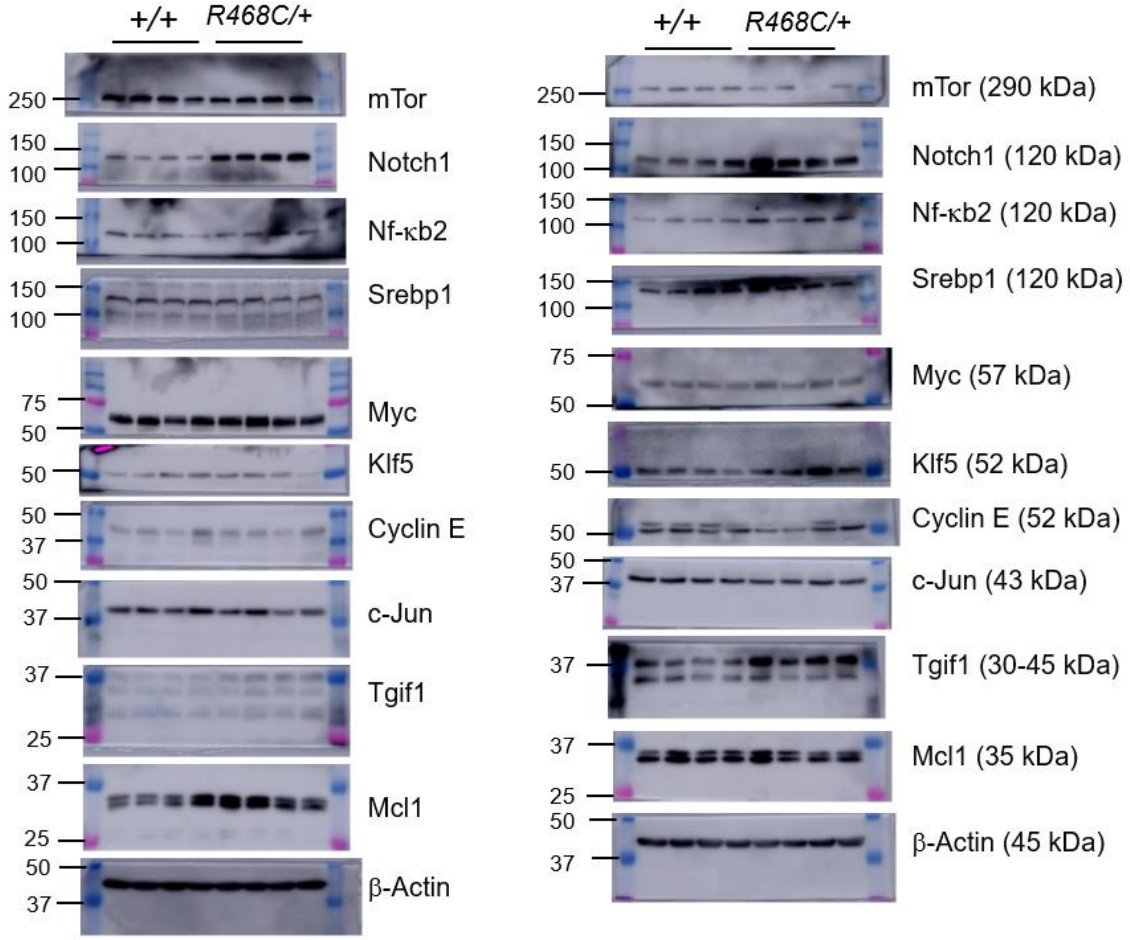

Supplementary Figure S4. Raw images of the blots in Figure 5.

Molecular weight markers and the molecular weight of each Fbxw7 substrate are shown.

Supplementary Table S1. Primers for qRT-PCR

Gene	Primer (5’-3’)	
c-Jun	Forward	TTTCTCACCAACTGCTTGGA
	Reverse	CCAAATGCTCCCCAAAATAC
Klf5	Forward	GGTCCAGACAAGATGTGAAATGG
	Reverse	TTTATGCTCTGAAATTATCGGAACTG
Myc	Forward	GCTGTAGTAATTCCAGCGAGAGACA
	Reverse	CTCTGCACACACGGCTCTTC
Notch1	Forward	CAATGTTCGAGGACCAGATGG
	Reverse	ACTGCAGGAGGCAATCATGAG
Tgif1	Forward	GAAACCCCAGCTTCACCTC
	Reverse	GCCAGATGCTGCAACAAG
Gapdh	Forward	CCCTTAAGAGGGATGCTGCC
	Reverse	TACGGCCAAATCCGTTCACA
